# Hepatitis C Virus Cryoglobulinemia and Non-Hodgkin Lymphoma

**DOI:** 10.5812/hepatmon.818

**Published:** 2012-02-29

**Authors:** Zohreh Jadali

**Affiliations:** 1 Department of Immunology, School of Public Health, Tehran University of Medical Sciences, Tehran, IR Iran

**Keywords:** Hepatitis C Virus, Cryoglobulinemia, Non-Hodgkin Lymphoma

## Abstract

**Context:**

On the strength of epidemiological data,biological studies, and clinical findings, hepatitis C virus appears to be involved in the pathogenesis of a proportion of patients with non-Hodgkin lymphoma and cryoglobulinemia.

**Objectives:**

The aim of this paper is to review the published literature focused on the current knowledge concerning hepatitis C virus and its potential role in the production of non-Hodgkin lymphoma and cryoglobulinemia in susceptible individuals.

**Evidence Acquisition:**

In this review, databases such as pubMed, embase, ISI, and Iranian databases including Iranmedex, and SID were searched.

**Results:**

The results of this review indicate that HCV infection may be a likely cause of various B cell dysregulation disorders such as non-Hodgkin lymphoma and cryoglobulinemia.

**Conclusion:**

Based on current findings, it has been hypothesized that NHL and cryoglobulinemia in HCV infection may have an immune-mediated pathogenesis. In HCV infected patients, we showed an elevated risk of these two diseases. These finding suggested a possible role for chronic hepatitis C in the pathogenesis of NHL and cryoglobulinemia.

## 1.Context

Hepatitis C virus (HCV) appears to be the virus that is usually associated with profound alterations in the host immune system, resulting in immunological abnormalities and some autoimmune disease [1][2][3]. It has been proposed that HCV infects and replicates in not only hepatocytes but also immune cells, such as B cells. HCV infection of B cells is the possible cause of B cell dysregulation diseases and conditions, including mixed cryoglobulinemia (MC), rheumatoid factor (RF) production, and B cell lymphoproliferative disorders that may progress to non-Hodgkin lymphoma (NHL) [4]. Interestingly,some patients develop low-grade lymphoma, composed of B cells that are immunophenotypically identical to the expanded B cells that are observed in cryoglobulinemia [5]. Most of the extrahepatic manifestations (EHMs) of HCV infection are associated with autoimmune or lymphoproliferative states and are thought to be immune-mediated. Patients with chronic hepatitis C infection (CHC) usually have immunological characteristics, such as circulating autoantibodies and deposits of immune complexes (ICs) in various tissues other than the liver, which may be an important pathogenic mechanism of extrahepatic disorders in the course of HCV infection [6]. HCV also appears to be capable of directly infecting cells around the liver—a feature that may play an important role in determining some EHMs.

This hypothesis is supported by observations of HCV replication in extrahepatic tissues, such as bone marrow, the central nervous system, endocrine glands, lymph nodes, spleen, monocytes, macrophages, and skin cells. Moreover, there is some epidemiological evidence of relationship between chronic HCV infection and some of these EHMs [7][8][9]. To date, the pathogenic mechanisms by which HCV acts as an instigator of B-cell lymphoproliferative disorders are not fully explained—the exact mechanisms by which B cells become dysregulated during the course of chronic HCV infection are not known. Diﬃculties in identifying disease mechanisms presumably stem from several issues, such as the wide heterogeneity of disease complications. Moreover, factors aﬀecting the development of the disease may be related to the host, the virus, and the environment [10].The purpose of this study was to review the literature concerning the association between HCV infection, cryoglobulinemia, and NHL.

## 2. Evidence Acquisition

### 2.1. Cryoglobulinemia

Cryoglobulins are immunoglobulins (Igs) that precipitate in the cold and are classiﬁed into three groups, based on Ig clonality [11][12][13][14]. Type I cryoglobulins are usually associated with lymphoproliferative disorders, including multiple myeloma and Waldenstrom macroglobulinemia, and usually consist of monoclonal Ig or a light chain. Type II cryoglobulins are composed of a polyclonal IgG and a monoclonal IgM RF reacting against the Fc portion of the IgG; it is identiﬁed as essential MC. It is believed that most of these essential MC are associated with CHC. Type III MC is also speciﬁed by RF activity, although polyclonal IgG and polyclonal IgM exist. There is a well-established link between type I cryoglobulinemia and well-known hematological diseases. This type of cryoglobulinemia is usually asymptomatic per se; similarly, circulating mixed cryoglobulins are commonly identiﬁed in a large number of infectious or systemic diseases [15]. The ﬁrst report indicating an association between HCV and cryoglobulinemia was published in 1990 [16]. However, the number of cases of cryoglobulinemia associated with HCV reported annually has risen considerably. The incidence of HCV infection in MC ranges from 40% to 90% and varies geographically [17][18]. MC can be observed in up to 60% of HCV-infected patients, although nearly 5% to 20% of these patients develop overt cryoglobulinemic syndrome [19]. This prominent association rate represents a causative link between HCV and MC [20].

However, HCV seems to play a prominent etiological role in the disease, especially in the Mediterranean area. Patients with HCV-negative MC are more frequently identiﬁed in the same areas where the overall prevalence of the disease is considerably low and where its relation with HCV is less common. These patients may represent true ‘essential’ MC [17][21]. Further evidence of an association between HCV infection and cryoglobulinemia has been provided by recent ﬁndings relating the presence of HCV RNA in the serum and/or peripheral mononuclear cells of patients with type II essential MC [22][23]. HCV antigens are detected within the ICs that precipitate in many organs, including the skin and kidneys of HCV-infected patients with MC—additional evidence in humans of a link between cryoglobulinemia and HCV infection [17].

### 2.2. phatophysiology of HCV-Related Cryoglobulinemia

HCV infection of peripheral lymphocytes suggests that this virus could be the stimulating factor for the lymphoproliferation that underlies MC. The mechanisms responsible for the clonal ampliﬁcation of autoantibody-producing B cells and chronic lymphoproliferation in HCV infection remain controversial. Nonetheless, it has now become obvious that HCV has high tropism for peripheral lymphocytes, which may act as its reservoir and a site of replication [24]. On B cells, CD81, which is part of a complex formed by CD21, CD19, and Leu13, lowers the threshold for B-cell activation. Flint et al. showed that HCV attaches to the CD81 ligand tetraspanin on the surface of B-lymphocytes through E2 protein (the second portion of the HCV envelope), resulting in activation of these lymphocytes [25]. Thus, the CD81 molecule is an HCV receptor, and HCV infection of B cells is the possible cause of diﬀerent disorders of B cell dysregulation, including MC and RF production [26].

It has also been shown that isolated intrahepatic B lymphocytes are able to spontaneously secrete RFs that usually exhibit the WA crossreactive idiotype. The occurrence of oligoclonal or monoclonal patterns indicates that B cells derived from very few or single cells expand within the liver. It is also possible that initially, only polyclonal cryoglobulins are produced, after which a dominant B-cell clone emerges, synthesizing monoclonal Igs [27][28]. Therefore, expansion of autoantibodyproducing B-cells, due to dysregulation of anti-idiotype networks, may be one of the possible mechanisms for disease induction. Molecular mimicry represents another potential mechanism of B-lymphocyte activation and autoantibody production. For example, it seems that molecular mimicry between HCV antigens, such as NS5A and HCV core proteins and host autoantigens, triggers activation of B-lymphocytes and autoantibody production [29]. Furthermore, the involvement of ICs has been suggested in the pathogenesis of disease. Various autoantibodies, including RF and cryoglobulins, are important in the formation of ICs [30]. Moreover, the importance of the complement system must not be forgotten, because complement components that may be generated in the course of an infection are involved in the immune complex-mediated reactions. This system is highly activated in cryoglobulinemic patients [31], and efﬁcient engagement of C1q protein by cryoglobulins may be the chief pathogenetic mechanism involved in the cryoglobulin-related pathway [32]. There is no doubt that dysregulation of helper T cells and their cytokines aids in the production of autoantibodies, such as cryoglobulins. Nonetheless, there is some controversy concerning the pattern of cytokines in patients with HCV and cryo-globulinemia. On one hand, Atta et al. veriﬁed that HCV carriers with cryoglobulinemia have increased serum levels of IL-5, a Th2 cell-derived cytokine [33]. Therefore, it is possible that Th2 responses that promote autoantibody production may responsible for the pathology of the disease. On the other hand, Saadoun et al. indicated that T cells from the liver of HCV-MC vasculitis patients display a signiﬁcantly augmented liver Th1 proﬁle compared to MC-negative controls [34]. Therefore, further experiments are needed to clarify the role of Th1/Th2 cytokines in the pathogenesis of the disease. Genetic factors also appear to play a role in the disease [35]. The high incidence of the disease among Mediterranean people and the association of certain human leukocyte antigen (HLA) speciﬁcities or alleles with disease have provided additional supports for this possibility. It seems that HLA type II polymorphisms, by interacting with T-cells, may inﬂuence the production of cryoglobulins [36][37]. In recent years, cryoglobulinemia has been a substantial focus, because it has become clear that cryoproteins may provoke signs and symptoms more often than previously identiﬁed. Mixed cryoglobulinemia symptoms (MCS) manifest in 10, 15% to 30% of MC patients and in 5, 10% of all HCV-infected subjects [38]. Due to the diverse clinical pictures, the presentation of MCS may signiﬁcantly diﬀer in various cases and in the same subject at various times. These clinical manifestations rely on the underlying disease; the dominant lesion of this disease is immune-mediated vasculitis. It may occur as a result of intravascular IC deposition in many organs, including skin, kidney, and peripheral nerves [22][39][40]. Other frequent symptoms of MCS are weakness, arthralgias, and palpable purpura that usually ﬁrst develops in the lower extremities [41]. Further signs may include Raynaud phenomenon, peripheral neuropathy, sicca syndrome, and membranoproliferative glomerulonephritis (MPGN), as well as lung disorders, fever, hematocytopenia, and diffuse vasculitis [42][43][44]. The optimal therapeutic strategy for HCV-associated MC is based on both antiviral and limited immunosuppressive therapy with regard to activity and severity of the underlying vasculitis and the status of the underlying infection [45]. IFN-α and ribavirin (RBV) are powerful antiviral drugs and can improve many of the clinical manifestations of MC, including skin vasculitis and renal disease [40]. In spite of its remarkable therapeutic properties, IFN-α may have unwanted eﬀects. For instance, Beuthien et al. introduced a case of a chronic HCV-infected patient who did not demonstrate signs of vasculitis or neuropathy before starting IFN therapy. This patient presented with cryoglobulinemic vasculitis with rigorous peripheral sensorimotor neuropathy following a virologic response that had been induced by antiviral treatment with pegylated interferon (PEG-IFN) and RBV [46]. In addition, deterioration of vasculitis in patients with cryoglobulins without any new onset of vasculitis has been described [47]. This observation has been conﬁrmed by other studies [48]. As a result, it is important for clinicians to be aware of these potentially severe side eﬀects.

### 2.3. Non-Hodgkin Lymphoma

In 1958, Denis Burkitt recognized a new type of lymphoma in Uganda, which was named Burkitt lymphoma [49]. In 1966, a classiﬁcation of lymphoid neoplasms was proposed by Rappaport. He subsumed these cases under the term “NHL” [49]. This disease is the most common cancer of the lymphatic system and is broadly divided into several subtypes, based on the predominant cell type of the tumor [50]. The exact cause of NHL is unknown, but it appears that the disease is related to many risk factors, such as:

Certain conditions that can also weaken the immune system, such as acquired immunodeﬁciency syndrome, and immunosuppressive medications, particularly after transplants [50].

Exposure to certain viruses, such as Epstein-Barr virus, human T-lymphotropic virus type 1, human immunodeﬁciency virus, HCV, and certain bacteria, such as Helicobacter pylori [50][51].

Family history of NHL—for example, having a sibling with the disease (though no hereditary pattern has been established) [52].

Exposure to chemicals, such as pesticides and solvents

[53]

A possible association between HCV infection and B-cell NHL (B-NHL) was ﬁrst reported in 1994 in Italy [54]. The results of this study demonstrated a signiﬁcant increase in the prevalence of HCV in a group of NHL patients (34%) compared with controls (1%). Subsequently, an association between HCV infection and B-NHL has also been noted by several epidemiological studies, indicating a high prevalence of HCV infection in B-NHL, ranging between 3.6% and 37% [55][56]. Similarly, the high prevalence of HCV infection in patients with B lymphoma provides further support of the link between HCV infection and B-cell NHL [57]. Additionally, there is a consistent association between HCV and MC. Chronic infection with HCV correlates with MC type II, which can progress into overt lymphoma in some patients [58]. In light of these data, a strong relationship between NHL and HCV infection is implicated, which may be associated with extra-nodal involvement (especially salivary glands and liver), splenomegaly, lymphocytosis, cryoglobulins, cytolysis, and colestasis [59][60][61]. The treatment strategy for HCV-associated NHL is similar to that of conventional lymphoma [62]. Among these therapeutic methods, which may be implemented alone or in combination, immunotherapy is a newer approach. Several immunotherapeutic methods have been proposed in clinical trials, and studies have indicated that IFN treatment is generally well tolerated [63]. With respect to NHL as an EHM of HCV infection, IFN-α appears to be an eﬀective treatment [63][64]. The response to IFN therapy for chronic hepatitis C has been improved tremendously with the use of RBV and PEG-IFN [63]. However, some patients do not respond to this therapy, and several factors can contribute to unre-sponsiveness, including the characteristics of the virus, the subject who has been infected, and the therapy [63]. In addition, IFN treatment has side eﬀects. These unwanted eﬀects can be divided into acute eﬀects (such as fever, chill and rigors, headaches, myalgias, and malaise) that decline over time and those that are chronic (including fatigue, anorexia, neuropsychiatric symptoms) [65]. Although antiviral therapy is an attractive therapeutic tool, a better understanding of the biology of IFN, its side eﬀect proﬁles, and toxicity management will help to optimize its application in the treatment of HCV-related NHL patients.

### 2.4. Pathophysiology of HCV-Related NHL

The possibility of a pathogenic link between HCV and NHL has been strengthened by the ﬁnding of HCV antigens in tissues from patients with NHL [66], although the pathogenic mechanisms through which the virus induce clonal proliferation and transformation of B-cells are not known. A recent retrospective study has shown that HCV is associated with NHL, as well as other B cell lymphoproliferative disorders, such as Waldenström macroglobulinemia and monoclonal gammopathy of unknown signiﬁcance [67]. Moreover, despite evidence of the presence of HCV in malignant cells [68][69], HCV viremia has been reported in up to 35% of patients with B cell lymphoma and nearly 90% of NHL patients with cryoglobulinemia [42]. The data also indicate that extrahepatic manifestations of HCV infection, such as high serum levels of RF activity, cryoglobulins, monoclonal gammopathy of undetermined signiﬁcance, and frank B-cell NHL, are virtually always associated with B-cell clonalities, which may have a direct impact on clinical features [70]. Further evidence to support the hypothesis that HCV is directly involved in the emergence and maintenance of B-cell expansion has been provided by a demonstration of HCV enrichment in intrahepatic inﬂammatory inﬁltrates [35]. This result is consistent with observations that HCV infects and may replicate in B cells [71][72][73][74][75] and lead to malignant transformation. However, this model can not describe the RF restriction that is seen in HCV MC, unless it is assumed HCV preferentially infects RF+ B cells. Nonetheless, a B cell line that productively releases infectious HCV has been demonstrated [74], and several groups have detected a negative HCV strand, a viral replicative intermediate, associated with lymphocytes from HCV+ patients [23][76][77]. Alternatively, it has been proposed that speciﬁc HCV proteins are essential for clonal B cell expansion. This suggestion is strengthened by the following considerations:

-IgG Fc binding activity of HCV core [78], which could result in enhanced HCV-IC generation.

-Abrogation of p53-induced apoptosis by the NS5A protein of HCV [79]

-Elevated expression of activation-induced cytidine deaminase in CHC B cells after E2-CD81 interaction.

Cytidine deaminase is also expressed in B-NHL patients [80][81][82] and may be required for germinal center-derived lymphomagenesis [83]. The E2-CD81 interaction could enhance the frequency of VDJ rearrangements in antigen-reactive B-cells [84][85][86], with possible Bcl-2 proto-oncogene activation [84][85]. Bcl-2 acts as an anti-apoptotic molecule, and its activation may be associated with the t (14;18) chromosomal translocation. Interestingly, this translocation has been observed repeatedly in B-cells of HCV-infected individuals, particularly in those with type t MC [84][87], and could lead to abnormal B-cell survival [87]. The extended survival of these highly selected B-cell clones may be related to the overexpression of genes (such as CCND1 and CCND2) in CHC B cells. This is an important ﬁnding, because enhanced expression of CCND, which changes cell cycle progression, has been frequently observed in diﬀerent tumors and may participate in tumorigenesis [88][89]. Additionally, CCND2 is known to be expressed at constitutively high levels in B-NHL patients [90] and thus may be associated with Blymphomagenesis. Recent ﬁndings also suggest that B cell survival that occurs in HCV MC [91][92][93][94] may be related to the elevated levels of B cell-activating factor. This molecule is a member of the tumor necrosis factor family and may play a role in the survival of autoreactive B cells.

## 3. Results

Since cryoglobulin production is associated with B lymphocyte expansion, it is possible that B-cell NHL may contribute to complications of MC syndrome, usually after a long-lasting follow-up period [42]. This occurrence, together with the remarkable association between MC and HCV infection, suggests a possible association between the same virus and ‘idiopathic’ B-cell NHL [56]. The latter possibility is partly supported by data showing the similarities that are shared by rearranged Ig genes in B cells from patients with type II MC and malignant B-cells from HCV-positive patients with B-cell NHL [95][96]. Based on the facts and interpretations above, it should be noted that lymphomathogenesis is a complex and multistep process, in which HCV may act as an antigenic trigger in addition to other environmental and genetic factors. In other words, some investigators believe that one of the outcomes of long-term HCV infection is clonal B cell expansion of Ig (cryoglobulin)-secreting lymphocytes. This process, which can be regarded as one of the suspected environmental factors, in combination with genetic factors, may culminate in a mutational event, with activation of oncogenes, resulting in NHL.

## 4. Conclusions

HCV infection has been associated with numerous EHMs. The majority of HCV chronically infected patients has no hepatic symptoms and may have normal alanine transaminase. In other words, sometimes, the importance of EHM is more severe than the hepatic disease itself. Therefore, EHMs may be the reason that patients seek medical care and thus have a chance to be studied, identiﬁed, and ﬁnally suitably treated. Some of these EHMs, such as cryoglobulinemia and NHL, are thought to be the result of a HCV/B-cell interaction that possibly predisposes one to B-cell proliferation and clonal expansion. The antigenic dependence of these B cells is demonstrated by evidence that HCV-related MC and NHL vanish after successful management of HCV infection. Whether B-cell clonal expansion of a particular speciﬁcity take places as a consequence of distinct selection is not obvious. The process of B-cell clonal expansion in a milieu that is favorable to the immortalization of one particular clone must be elucidated. Predisposing elements for transforming events must be recognized.
